# Supplementary material for: Secondary or Specialized Metabolites, or Natural Products: A Case Study of Untargeted LC–QTOF Auto-MS/MS Analysis
Source: Cells. 2022 Mar 17;11(6):1025. doi: 10.3390/cells11061025 (PMC8963255; doi:10.3390/cells11061025)
Supplement: Supplementary file 1 [file cells-11-01025-s001.zip › Supplements/Figure S1.pdf]

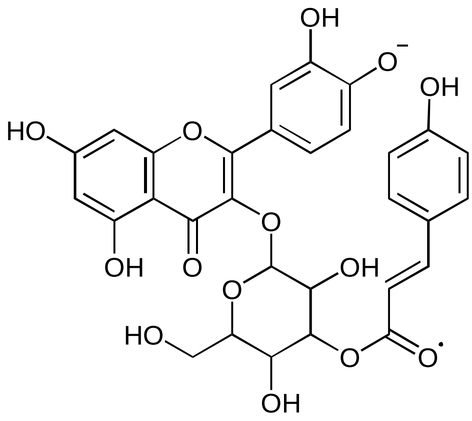

Chemical Formula: $C_{30}H_{25}O_{14}^{\bullet-}$
Exact Mass: 609.1250

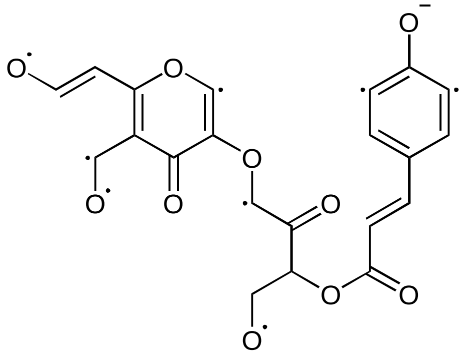

Chemical Formula: $C_{21}H_{11}O_{10}^{8\bullet-}$
Exact Mass: 423.0358

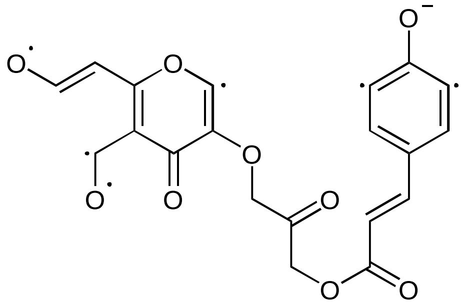

Chemical Formula: $C_{20}H_{11}O_{9}^{6\bullet-}$
Exact Mass: 395.0409

Figure S1. In-source and MS/MS fragmentation of quercetin-3-(3''-p-coumaroyl)-glu-rha (9.11 min)
